# Two-Parametric Immunological Score Development for Assessing Renal Involvement and Disease Activity in Systemic Lupus Erythematosus

**DOI:** 10.1155/2018/1294680

**Published:** 2018-08-30

**Authors:** Christopher Sjӧwall, Chelsea Bentow, Mary Ann Aure, Michael Mahler

**Affiliations:** ^1^Department of Clinical and Experimental Medicine, Rheumatology/Division of Neuro and Inflammation Sciences, Linköping University, Linköping, Sweden; ^2^Research and Development, Inova Diagnostics, San Diego, CA, USA

## Abstract

**Objective:**

Anti-double-stranded (ds) DNA and anti-C1q autoantibodies are useful tools in the assessment of disease activity and nephritis in systemic lupus erythematosus (SLE) patients. This study aimed to explore the utility of these antibodies along with anti-Ku antibodies in an oligoparametric model approach for the assessment of disease activity and lupus nephritis.

**Methods:**

Samples from 261 well-characterized SLE patients were tested using chemiluminescent immunoassays (CIA) for anti-dsDNA and anti-Ku antibodies as well as by anti-C1q antibody ELISA (Inova Diagnostics, USA). Of these SLE patients, 26.4% had lupus nephritis (LN) at the time of blood draw or had a history of LN, and modified SLE disease activity index-2K (SLEDAI) scores were used to assess disease activity.

**Results:**

All three antibodies demonstrated higher prevalence and higher antibody levels in active versus inactive SLE patients and in LN versus non-LN patients. When oligoparametric analysis was performed, the likelihood of LN and patients with active disease increased with dual and triple positivity.

**Conclusions:**

Anti-dsDNA and anti-C1q antibodies are useful tools to identify disease activity and/or renal involvement in SLE patients. In addition, the combination of those antibodies in a two-parametric score might improve the clinical utility of those markers.

## 1. Introduction

Combinations of clinical manifestations and symptoms of systemic lupus erythematosus (SLE) can vary widely among affected patients, and appropriate management is thus critically dependent upon the proper assessment of disease activity (DA) [1, 2] and damage accrual [3]. Although antibodies such as anti-double-stranded (ds) DNA and anti-C1q antibodies [3–8] as well as urinalysis and complement consumption [9] have been shown to correlate with DA [2, 10, 11] and the likelihood of renal involvement in lupus patients [1, 3], the additive effect of combined biomarkers is not widely implemented. In addition, recently anti-Ku antibodies, that have mostly been described in the context of idiopathic inflammatory myopathies, have now been reported to be associated with SLE [12]. However, it is yet unclear whether anti-Ku-positive SLE cases show a higher degree of myositis or other musculoskeletal manifestations. The Ku autoantigen is a heterodimeric protein comprised of two subunits (Ku70 and Ku80) with sequence-specific binding affinity for DNA and to a lesser extent for RNA [13]. Present in most eukaryotic cells, Ku is an abundant nuclear protein that contains ssDNA-dependent ATPase and ATP-dependent DNA helicase activities representing the regulatory subunit of the DNA-dependent protein kinase that phosphorylates many proteins, including SV-40 large T antigen, p53, RNA-polymerase II, RP-A, topoisomerases, hsp90, and many transcription factors. The multifunctional protein has been directly or indirectly implicated in many important cellular metabolic processes such as repair of double-stranded DNA breaks, V (D) J recombination of immunoglobulins and T-cell receptor genes, immunoglobulin isotype switching, DNA replication, transcription regulation, regulation of heat shock-induced responses, regulation of the precise structure of telomeric termini, and a novel role in G2 and M phases of the cell cycle.

Using a cross-sectional cohort of well-characterized Swedish SLE patients, this study investigated the utility of an SLE assessment model using a combination of biomarkers, namely, anti-dsDNA, anti-C1q, and anti-Ku antibodies.

## 2. Materials and Methods

### 2.1. Patient Characteristics and Samples

In this study, blood samples from 261 well-characterized patients (Table 1) diagnosed with SLE were included and tested using chemiluminescent immunoassays (CIA) for anti-dsDNA and anti-Ku (research use only) antibodies as well as by anti-C1q antibody ELISA (all methods Inova Diagnostics, San Diego, USA). All patients took part in the prospective, structured follow-up program “KLURING” (Swedish acronym for *Clinical LUpus Register In Northeastern Gothia*) [14, 15], including registration of disease phenotypes, ongoing medication, and comorbidities, at the Rheumatology outpatient clinic, Linköping University Hospital, Sweden. The vast majority of the patients (241/261), that is, 92.3% met at least 4 of the revised 1997 American College of Rheumatology classification criteria (ACR-97) [16]. Another 20 patients (7.7%) fulfilled the 2012 Systemic Lupus International Collaborating Clinics (SLICC) classification criteria [17] without meeting ACR-97. The patients were recruited consecutively. Most were established cases (194 patients, 74.3%), but 67 (25.7%) had recent-onset disease at the time of sampling. Of these 261 SLE patients, 69 (26.4%) had active lupus nephritis (LN) or had the history of LN, defined by the ACR classification criterion at the time of blood draw. The presence of APS-associated nephropathy was registered and defined by histology characterized by acute thrombotic lesions in glomeruli and/or arterioles (thrombotic microangiopathy) or more chronic vascular lesions [18, 19]. The SLE disease activity index-2K (SLEDAI) [20] scores were available for all patients, and the index was also modified with the exclusion of serological items (mSLEDAI) for all analyses comparing DA and anti-dsDNA (CIA). To discriminate serologically active but clinically quiescent patients from clinically active cases, a SLEDAI cut-off of ≥5 was used to define active disease (37/261, 14.2% active). Regarding serological items included in SLEDAI, the *Crithidia luciliae* indirect immunofluorescence test (CLIFT) instead of the proposed Farr assay was used to detect anti-dsDNA antibody binding and nephelometry to measure levels of complement proteins C3 and C4 [2, 20]. The term “active LN” was defined as any positive item of the four renal subcomponents (urinary casts, hematuria, proteinuria, or pyuria) of SLEDAI (renal score 4–16), whereas the term “inactive LN” was defined as absence of the above. Ongoing SLE medication and acquired organ damage according to the SLICC/ACR damage index score (SDI) [21] was registered at the time point of blood sampling.

Peripheral venous blood was drawn from each individual. Serum was prepared and stored at −70°C until analyzed. At all patient visits, routine laboratory analyses such as leukocytes, erythrocytes, platelets, urinalysis, CRP, complement proteins, and erythrocyte sedimentation rate (ESR) were performed at the Clinical Chemistry unit at Linköping University Hospital. Oral and written informed consent was obtained from all subjects.

### 2.2. Statistical Analysis

All statistical analyses were performed by *Analyse-it*® for Excel method evaluation software (version 3.90.1; Leeds, UK). Likelihood plots were used to assess the association of the different markers for the association with disease activity and LN. The oligoparametric analysis was performed at both the manufacturer's cut-off for the methods and the optimized cut-off points based on likelihood plots to increase the odds ratio (OR). Differences between subgroups were calculated with Mann–Whitney *U* test or Fisher's test or chi-square test (where appropriate).

## 3. Results

As demonstrated in Table 1, patients with active disease (*n* = 37) were more often males and of non-Caucasian ethnicity; they were significantly younger, had shorter duration of SLE, and were more likely to have suffered from oral ulcers and lupus nephritis compared to the inactive cases (*n* = 224). In addition, active patients used higher daily doses of prednisolone and were more often on mycophenolate mofetil and antihypertensive therapy in comparison with the inactive cases.

**Figure 1:**
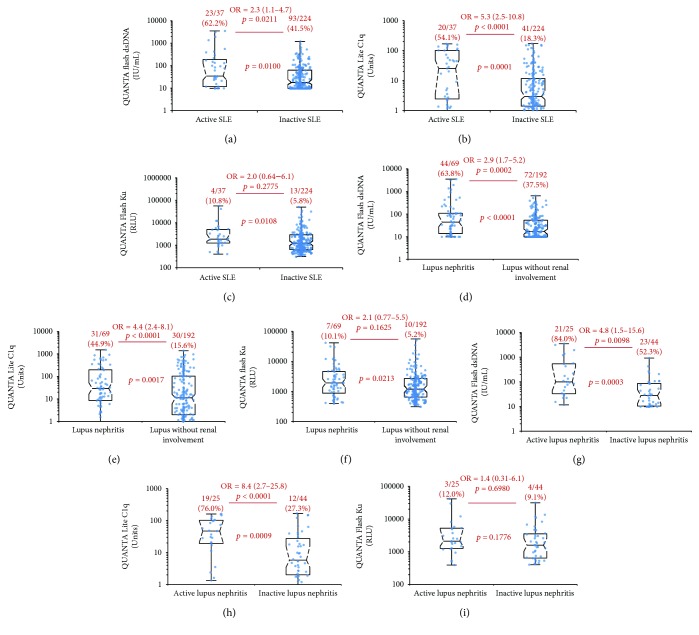
Comparative antibody titer distribution of anti-dsDNA, anti-C1q, and anti-Ku antibodies among patients with active versus inactive SLE (a–c), lupus nephritis versus lupus without renal involvement (d–f), and active versus inactive nephritis (g–i). The prevalence of each marker and the significance level of the comparisons between groups are indicated in red text. The acronyms IU/mL and RLU stand for international units/mL and relative light units, respectively, which are assay unit values generated from the QF CIA technology.

**Figure 2:**
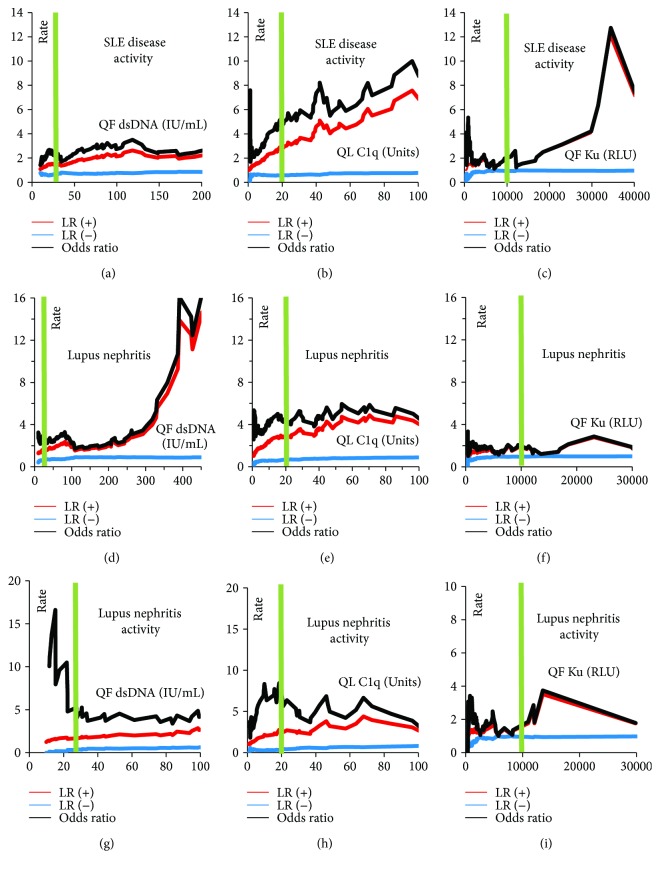
Likelihood plots for the assessment of disease activity, lupus nephritis, and lupus nephritis activity; likelihood ratios are shown as a function of antibody titer. Likelihood plots of the QUANTA Flash (QF) dsDNA, QUANTA Lite (QL) C1q, and anti-Ku chemiluminescent immunoassay (CIA) for disease activity (a–c), lupus nephritis (d–f), and lupus nephritis activity (g–i). The black line indicates the odds ratios (OR), while the red and blue lines denote the positive and negative likelihood ratios, respectively. Furthermore, the green vertical line represents the manufacturer's cut-off point. The acronyms IU/mL and RLU stand for international units/mL and relative light units, respectively, which are assay unit values generated from the QF CIA technology.

**Figure 3:**
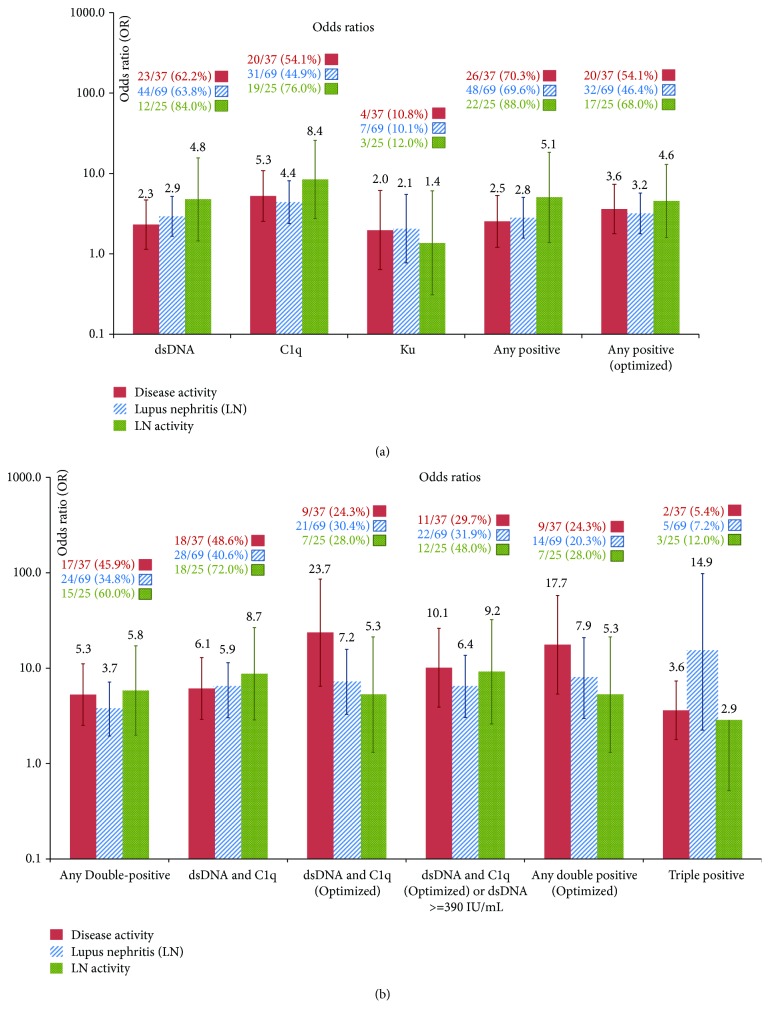
Association of single markers anti-dsDNA, anti-C1q, and anti-Ku antibodies (a) and oligoparametric models (b) with disease activity, lupus nephritis, and lupus nephritis activity. The number of patients and percentage of positive patients are indicated above the graph. The *y*-axis is shown in logarithmic scale. Error bars indicate the 95% confidence intervals. The term *optimized* pertains to a use of an alternative cut-off (other than the manufacturer's cut-off) which gave the best likelihood ratios in Figure 2.

**Figure 4:**
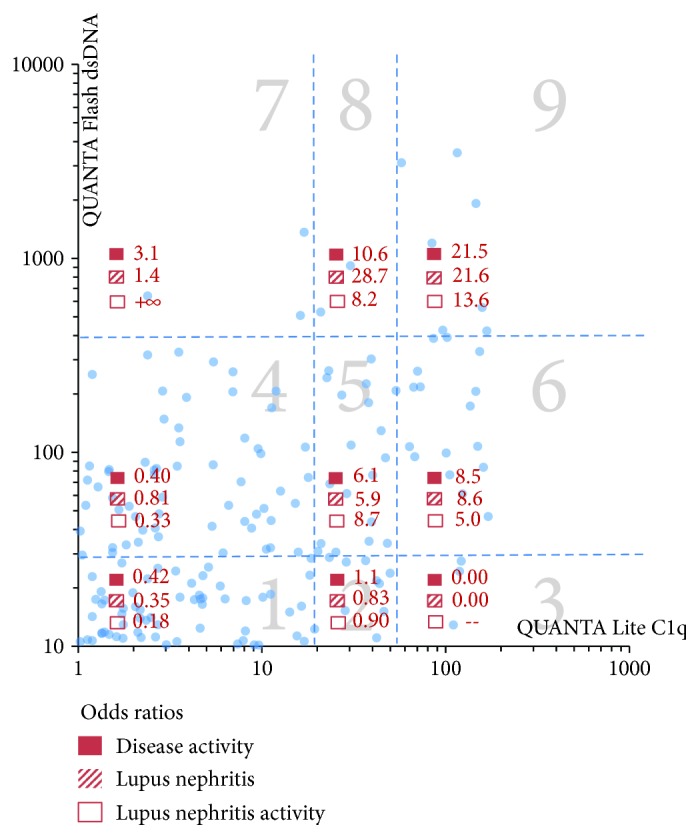
Anti-dsDNA and anti-C1q combined odds ratios for the assessment of disease activity, lupus nephritis, and lupus nephritis activity. The dotted lines sectioning the graphs into 9 groups represent different cut-off selections including the manufacturer's cut-off points at 27 IU/mL for anti-dsDNA and 20 units for anti-C1q antibodies and optimized cut-off points at 390 IU/mL for dsDNA and 54 units for C1q. The different sections of the graph are indicated by numbers (1–9). The *x* and *y*-axes are shown in logarithmic scale. The symbol (--) stands for odds ratios that cannot be calculated due to patients not having values within the range of the respective quadrant.

In the total cohort, all three antibodies (anti-dsDNA, anti-C1q, and anti-Ku) demonstrated markedly higher prevalence and higher antibody levels in active versus inactive SLE patients, LN versus non-LN patients, and active versus inactive LN patients (Figure 1). When analyzing the prevalence of the antibodies statistically in active versus inactive disease, the ORs were 2.3 for dsDNA (*p* = 0.0211), 5.3 for anti-C1q (*p* < 0.0001), and 2.0 for anti-Ku antibodies (*p* = 0.2775). The antibody titers of the three antibodies were significantly higher in active versus inactive patients (*p* = 0.0100 for anti-dsDNA, *p* = 0.0001 for C1q, and *p* = 0.0108 for Ku). Similarly, when analyzing the prevalence of the antibodies in patients with and without LN, the ORs were 2.9 for dsDNA (*p* = 0.0002), 4.4 for anti-C1q (*p* < 0.0001), and 2.1 for anti-Ku antibodies (*p* = 0.1625). Likelihood plots were generated to observe the change of OR at different cut-off points for the three assays and note if any optimal cut-offs with higher OR could be observed (Figure 2). Along with the manufacturer's cut-off, different optimized cut-offs were selected based on the plots in Figure 2 to perform oligoparametric analysis when combining the markers. When oligoparametric analysis was performed by combining biomarker results, the likelihood of LN and patients with active disease increased with dual positivity and triple positivity (Figure 3). Combining different markers increased the OR in most cases but also reduced the number of patients that could be assessed with the score. Regression analysis was performed to compare the titers of anti-dsDNA and anti-C1q and calculate the ORs for LN and disease activity at different thresholds (Table 2 and Figure 4). When analyzing the antibody prevalence to clinical parameters of assessing renal function, the antibody positivity in the SLE cases with chronic kidney disease (CKD) class 4 or 5 (eGFR < 30) found positivity in 20.0% (anti-dsDNA), 0.0% (anti-C1q), and 0.0% (anti-Ku). Numerically, more patients with inactive disease had APS nephropathy compared to active cases (4 versus 1). Anti-dsDNA was positive in 2/5 (40.0%) of these cases, while anti-C1q and anti-Ku were not positive in any of the five cases (0.0%). However, the two anti-dsDNA positive APS nephropathy cases were in the equivocal range of the anti-C1q assay used.

## 4. Discussion

This study verifies the correlation of anti-dsDNA [1] and anti-C1q autoantibodies, [7, 22] with DA in SLE and LN. In addition, the data demonstrate the utility of a two-parametric model approach using biomarkers for assessing SLE patients for more active and severe disease, especially for patients that have, had a history of, or may develop LN. Furthermore, the results hold promise for the benefit of combined autoantibody profiling for the clinical management of patients [23].

In the single-variate analyses, anti-C1q antibodies using the standard cut-off value showed the highest OR both for active disease and for LN when using the cut-off proposed in the direction insert of the assays. This is in line with previous studies showing high clinical value in the monitoring of LN patients [3–7, 11, 24–26]. However, when considering the titer of the antibodies, anti-dsDNA antibodies when present at high titers showed very high OR for LN (at 390 CU, OR = 16). In contrast, no significant effect of the titer of anti-C1q antibodies on the presence or absence of LN could be observed. Whether antibodies against dsDNA and C1q are merely epiphenomena or if they are truly involved in the pathogenesis of SLE has been discussed. Indeed, anti-dsDNA and anti-C1q are frequently found both in serum and in inflammatory lesions in glomerulonephritis [27–29]. The fact that circulating antibody levels often correlate with DA and renal involvement has strengthened the assumption of pathogenetic importance of anti-dsDNA and anti-C1q in LN [30, 31]. When analyzing the antibody prevalence to clinical parameters of assessing LN activity, the antibody positivity in the SLE cases with CKD class 4 or 5 (eGFR < 30) demonstrated that the markers are not only a reflection of renal function since the positivity rate was low (anti-dsDNA 20.0%, anti-C1q 0.0%, and anti-Ku 0.0%). However, the number of patients in this CKD category was low (*n* = 5), and there may be other reasons aside from lupus that contributed to the renal failure in these patients. We did not have access to data on renal damage according to KDIGO 2012 [32], which constitutes a limitation of the study.

Although we found a weak association between anti-Ku antibodies and disease activity as well as LN, anti-Ku antibodies did not add much value to the combination of anti-dsDNA and anti-C1q antibodies. In addition, they were only found in a subpopulation of patients. Therefore, it is unlikely that anti-Ku antibodies will be used in the assessment of disease activity as well as LN and were also excluded from our cluster models. Instead, anti-Ku antibodies might be a more useful marker to diagnose patients with overlap syndromes, in particular with myositis [12].

In the era of precision medicine [23], biomarkers and biomarker combination will facilitate the stratification and management of SLE patients [33, 34]. Most likely, this approach will include genetic [33], proteomic, and metabolomic markers next to autoantibodies.

## Figures and Tables

**Table 1:** Detailed characteristics of the 261 SLE patients in relation to disease activity.

Patient characteristics	Mean (range) or %			*p* value^#^
	*All* (*n* = 261)	*Active* (*n* = 37)	*Inactive* (*n* = 224)	
*Background variables*				
Females (*n*)	86.6 (226)	75.7 (28)	83.0 (198)	<0.0001
Age (years)	49.2 (18–88)	43.2 (18–80)	50.3 (18–88)	0.02
Disease duration (years)	15.0 (0–45)	9.4 (0–32)	16.0 (0–45)	<0.0001
Caucasian ethnicity (*n*)	90.8 (237)	83.8 (31)	92.0 (206)	<0.0001
Ongoing tobacco smoking (*n*)	9.9 (26)	5.4 (2)	10.7 (24)	n.s.
SLICC/ACR damage index (score)	1.3 (0–9)	1.0 (0–7)	1.4 (0–9)	n.s.
SLEDAI (score)	2.9 (0–24)	11.0 (5–24)	1.6 (0–4)	<0.0001
mSLEDAI (score)	2.0 (0–20)	9.3 (5–20)	0.8 (0–4)	<0.0001
Estimated glomerular filtration rate (mL/min/1.73 m^2^)	84.0 (5–225)	91.4 (14–225)	82.8 (5–201)	n.s.
*Ongoing medication*				
Prednisolone dose (mg)	6 (0–60)	17.4 (0–60)	4.1 (0–50)	<0.0001
Antimalarials (%, *n*)	60.5 (158)	62.2 (23)	60.2 (135)	n.s.
Azathioprine (%, *n*)	5.7 (15)	5.4 (2)	5.8 (13)	n.s.
Cyclophosphamide (%, *n*)	0.8 (2)	5.4 (2)	0 (0)	∞
Cyclosporin (%, *n*)	1.5 (4)	0 (0)	1.8 (4)	∞
Methotrexate (%, *n*)	9.6 (25)	8.1 (3)	9.8 (22)	n.s.
Mycophenolate mofetil (%, *n*)	12.3 (32)	27.0 (10)	9.8 (22)	0.003
Rituximab (%, *n*)	3.1 (8)	8.1 (3)	2.2 (5)	n.s.
Sirolimus (%, *n*)	3.1 (8)	0 (0)	3.8 (8)	∞
Tacrolimus (%, *n*)	0.8 (2)	2.7 (1)	0.4 (1)	n.s.
Warfarin (%, *n*)	17.2 (45)	13.5 (5)	17.9 (40)	n.s.
Acetylsalicylic acid (%, *n*)	26.1 (68)	18.9 (7)	27.2 (61)	n.s.
Statins (%, *n*)	19.9 (52)	21.6 (8)	19.6 (44)	n.s.
*Clinical phenotypes (ACR-97 definitions)*				
Malar rash (%, *n*)	43.3 (113)	48.6 (18)	42.4 (95)	n.s.
Discoid rash (%, *n*)	15.7 (41)	13.5 (5)	16.1 (36)	n.s.
Photosensitivity (%, *n*)	49.0 (128)	51.4 (19)	48.7 (109)	n.s.
Oral ulcers (%, *n*)	11.9 (31)	27.2 (10)	9.4 (21)	0.002
Arthritis (%, *n*)	77.4 (202)	78.4 (29)	77.2 (173)	n.s.
Serositis (%, *n*)	39.1 (102)	27.0 (10)	41.1 (92)	n.s.
Renal disorder (%, *n*)	26.4 (69)	51.4 (19)	22.3 (50)	0.0002
Neurologic disorder (%, *n*)	5.4 (14)	8.1 (3)	4.9 (11)	n.s.
Hematologic disorder (%, *n*)	59.0 (154)	70.3 (26)	57.1 (128)	n.s.
Immunologic disorder (%, *n*)	49.4 (129)	62.2 (23)	47.3 (106)	n.s.
IF-ANA (%, *n*)	98.5 (257)	94.6 (35)	99.1 (222)	n.s.
*Other characteristics*				
Hypertension (%, *n*)	26.1 (68)	48.6 (18)	22.3 (50)	0.0007
APS-associated nephropathy^∗^ (%, *n*)	1.9 (5)	2.7 (1)	1.8 (4)	n.s.
Diabetes mellitus (%, *n*)	5.4 (14)	0 (0)	6.3 (14)	∞
End-stage renal disease (%, *n*)	3.1 (8)	5.4 (2)	2.7 (6)	n.s.

ACR = American College of Rheumatology; APS = antiphospholipid syndrome; IF-ANA = immunofluorescence microscopy antinuclear antibodies; n.s. = not significant; SLEDAI = systemic lupus erythematosus disease activity index 2000; mSLEDAI = modified SLE disease activity index; SLICC = Systemic Lupus International Collaborating Clinics. ^∗^Presence of APS-associated nephropathy was defined by histology characterized by acute thrombotic lesions in glomeruli and/or arterioles (thrombotic microangiopathy) or more chronic vascular lesions [18, 19]. ^#^Performed with Mann–Whitney *U* test or Fisher's test or chi-square test (where appropriate).

**Table 2:** Odds ratios (ORs) when combining anti-dsDNA and anti-C1q antibodies for the association with lupus nephritis (LN), LN activity and disease activity. Thresholds are based on clustering sections described in Figure 4.

Sections	Disease activity odds ratios (95% CI)	Lupus nephritis odds ratios (95% CI)	Lupus nephritis activity odds ratios (95% CI)
Cluster sections 1–4	0.47 (0.23–0.94)	0.35 (0.20–0.61)	0.27 (0.09–0.85)
Cluster sections 5–7	6.3 (3.0–13.3)	5.6 (2.9-10.7)	10.8 (3.4–33.8)
Cluster sections 8 and 9	10.6 (3.0–37.3)	28.7 (4.5–178.1)	8.2 (1.7–38.1)

## Abbreviations

ACR: American College of Rheumatology
CIA: Chemiluminescent immunoassay
CLIFT: *Crithidia luciliae* indirect immunofluorescence test
DA: Disease activity
IIF: Indirect immunofluorescence
LN: Lupus nephritis
SLE: Systemic lupus erythematosus
SLEDAI: SLE disease activity index-2K
mSLEDAI: Modified SLE disease activity index-2K
SLICC: The Systemic Lupus International Collaborating Clinics.
